# Effects of Interface Morphology on the Shear Mechanical Properties of Sand–Concrete Interfaces

**DOI:** 10.3390/ma16186122

**Published:** 2023-09-08

**Authors:** Huanhuan Li, Zhigang Meng, Songlin Shen

**Affiliations:** 1 School of Civil Engineering and Architecture, NingboTech University, 1 Qianhu South Road, Ningbo 315100, China; 2 Institute of Geotechnical Engineering, Zhejiang University, 866 Yuhangtang Road, West Lake District, Hangzhou 310058, China; 3 Shaanxi Key Laboratory of Safety and Durability of Concrete Structures, Xijing University, 1 Xijing Road, Xi’an 710123, China; 4 China MCC22 Group Corporation Ltd., 16 Xingfu Road, Tangshan 063000, China

**Keywords:** interface morphology, sand–concrete interface, shear mechanical behavior, shear strength, shear mechanism

## Abstract

The morphology of the contact surface between cast-in-place engineering structures and soil is generally random. Previous research focusing on the shear mechanical properties of soil–concrete interfaces has predominantly concentrated on the role of interface roughness by constructing regular concrete surface types, largely neglecting the potential impact of the roughness morphology (i.e., the morphology of the concrete surface). In this study, concrete blocks with the same interface roughness and different roughness morphologies were constructed based on the sand-cone method, including random rough surface, triangular groove surface, rectangular groove surface, trapezoid groove surface, and semicircular groove surface. A series of direct shear tests were conducted on the rough and smooth sand–concrete interfaces, as well as on natural sand. Through these tests, we examined the shear mechanical behavior and strength of the sand–concrete interfaces, and analyzed the underlying shear mechanisms. The results showed that: (i) the interface morphology had little effect on the variation in the shear stress–displacement curve of sand–concrete interfaces, and it had a significant influence on the shear strength of the interfaces; (ii) under the same normal stress, the shear strength of the sand–concrete interfaces with a random rough surface was the greatest, followed by the triangular groove surface, while the shear strength of the rectangular groove surface proved the lowest; (iii) the shear strength of the sand–concrete interfaces with the same roughness was affected by the size of the contact area between the concrete plane and the sand, that is, a larger contact area correlated with a decrease in shear strength. It can be concluded that the shear strength value of a sand–concrete surface with the triangular groove is the closest to the shear strength of a random rough interface. By gaining a deep understanding of the effects of different contact surface morphologies on shear strength and shear behavior, significant insights can be provided for optimizing engineering design and enhancing engineering performance.

## 1. Introduction

Soil–structure interaction features are among the keen research topics in geotechnical engineering, with significant achievements in investigating the mechanical features of soil–structure interfaces, such as studying the damage constitutive model of the soil–structure interface [1] and the effect elements on interfacial shear strength [2,3]. For the purposes of relevant studies, the structures were usually simplified into concrete slabs, steel plates, and other configurations [4,5], which were allocated different roughness surfaces before being subjected to a series of direct shear tests to explore the shear mechanical behavior and shear strength of these soil–structure interfaces [6]. The soil samples were produced from a varied range of materials, which primarily consisted of sand, clay, gravel-soil, loess, and other special soil materials [7]. The effects of soil moisture content, sand particle size, and sand relative density on the shear strength of the interfaces were explored and discussed [8,9]. The shear strength of the soil–structure interfaces was found to decrease with the increase in water content [10]. In addition, variables such as load conditions and interface roughness were also shown to affect the shear behavior and shear strength of soil–structure interfaces [11]. Maghsoodi et al. (2020) [12] proposed that whereas applying thermal loads had a negligible effect on the shear strength of sand–structure interfaces, increasing them did enhance the shear strength of clay–structure interfaces. Elsewhere, scholars reported that the shear strength of soil–structure interfaces rose with the increase in surface roughness of the structure [13,14]. It is fair to assert that most of the relevant studies to date have focused on investigating the effects of surface roughness on shear mechanical properties of soil–structure interfaces, neglecting other morphological parameters such as concave-convex shape, periodic structures, and other characteristics that are part of the interface morphology. These morphology features have a significant impact on friction, shear transmission, and mechanical behavior between interfaces. It is important to point out that the construction methods of such rough surfaces in the literature were not consistent, including both random rough surfaces and regular rough surfaces, in addition to which the surface roughness morphology was also inconsistent: their composition varied between triangular groove, rectangular groove, semicircular groove, and convex rhombus designs [15,16]. The authors had previously explored the influence of the inclined angle of the triangular groove method on the shear mechanical behavior of the soil–structure contact surface [17], but not the effects of the concrete surface morphology on the same.

A deeper understanding and analysis of the effects of different morphology characteristics on the shear mechanical properties of interfaces can help us gain better insights into the properties of real engineering interfaces and provide more rational guidance and optimization strategies for engineering design and construction. Therefore, investigating the impact of interface morphology characteristics on the shear mechanical properties of interfaces holds significant theoretical and practical significance. In the present work, we designed five distinct rough-morphology concrete surfaces using the sand-cone method, each with the same degree of roughness. The designs comprised random surfaces, rectangular groove surfaces, triangular groove surfaces, semicircular groove surfaces, and trapezoidal groove surfaces. A series of direct shear tests on sand–concrete interfaces at three relative densities were conducted. The shear stress displacements on contact surfaces with varying roughness morphologies and relative densities were analyzed. Additionally, the influences of different roughness morphologies on the shear strength of the soil–concrete interface under the three relative densities were investigated. The shear failure mechanism of different roughness morphology concrete surfaces was revealed.

## 2. Materials and Methods

### 2.1. Testing Equipment and Materials

**Figure 1:**
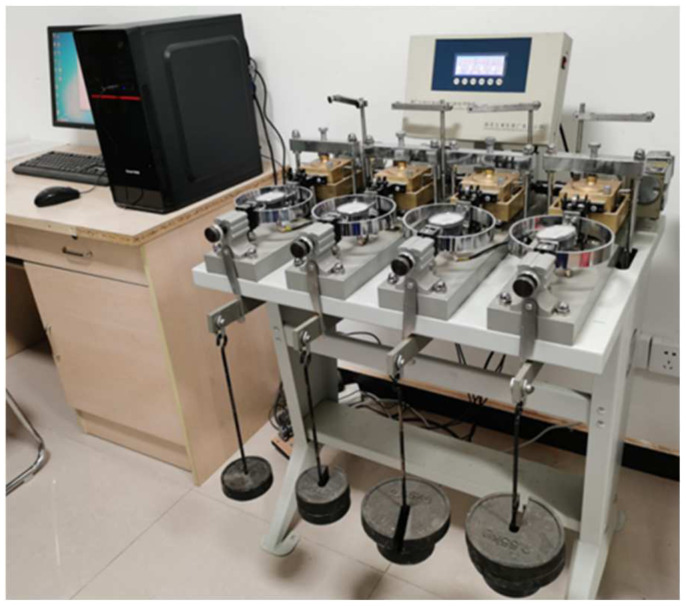
The ZJ strain controlled direct shear apparatus.

**Figure 2:**
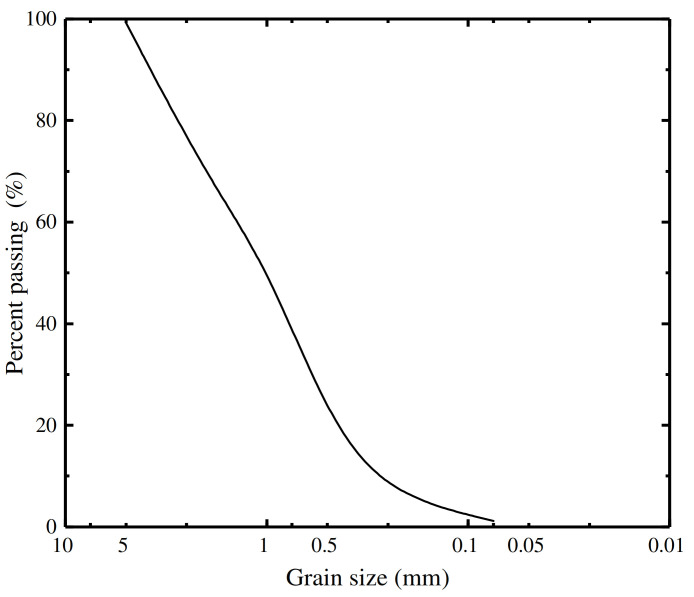
Particle size distribution curve of the test sand.

The ZJ strain controlled direct shear apparatus produced by Nanjing Soil Instrument Factory Co., Ltd. (Nanjing, China), was adopted for the direct shear tests (Figure 1). Natural river sand was used for the tests. According to the Geotechnical Test Method Standard GBT 50123-2019 [18], the specific gravity was measured based on the pycnometer method, the particle distribution was measured based on the sieve method, and the maximum (minimum) dry density was measured based on dry density tests, which were used to calculate the minimum (maximum) void ratio. The basic physical properties of the natural river sand sourced for the study are shown in Table 1. The value of the uniformity coefficient was 4.63, and that of the curvature coefficient was 0.89; thus, the test sand was determined to be poorly graded. The natural river sand, dried and made into sand samples with a saturation of 60%, was used for direct shear testing. Figure 2 shows the particle size distribution curve of the test sand. The concrete mix proportion was 0.5:1:2.11:2.53 for water, cement, sand, and stone. The P·O 42.5R cement used in this test was produced by Shaanxi Qinling Cement (Group) Co., Ltd. (Tongchuan, China).

### 2.2. Specimen Preparation

**Figure 3:**
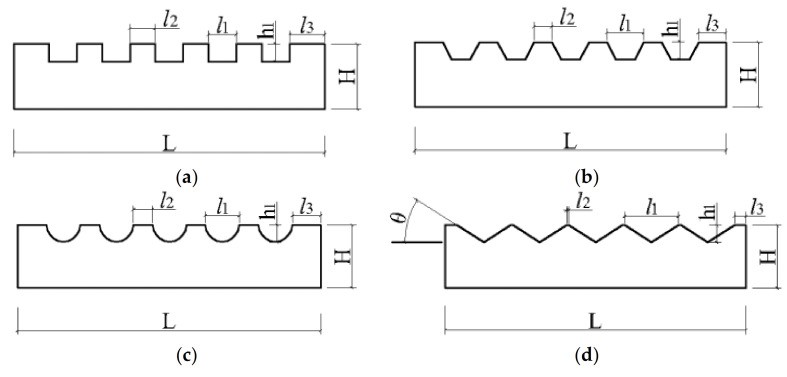
Diagrams of grooves with different morphologies: (**a**) rectangular; (**b**) trapezoid; (**c**) semicircular; and (**d**) triangular.

**Figure 4:**
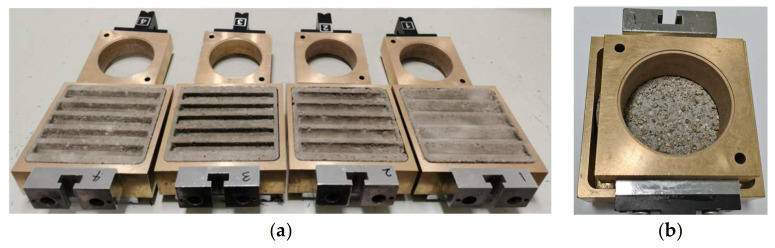
Direct shear boxes with concrete test blocks: (**a**) regular concrete surfaces; and (**b**) random concrete surface.

Based on the slurry intrusion test in sand, concrete blocks with random roughness surfaces were obtained [17]. The roughness of the random concrete surface was evaluated as 2.64 mm based on the sand cone method, which evaluates the roughness of the concrete surface through the average depth of the groove [17]. Based on the roughness value of the random concrete surface, concrete blocks with rectangular, trapezoid, semicircular, and triangular surface morphologies (collectively referred to as regular concrete surfaces) were produced separately. The surface morphology is shown in Figure 3. The specific dimensional parameters are listed in Table 2. The maximum size of the test blocks was determined according to the internal size of the lower box of the direct shear box so that the concrete block could be placed into the shear box (Figure 4a). Furthermore, smooth surface concrete blocks were also prepared.

### 2.3. Test Procedure

Three relative densities of sand samples (*D*r = 30%, 50%, 70%, respectively) were designed for the direct shear test, and each relative density corresponded to 7 sets of shear tests, as shown in Table 3. The four normal stresses, *σ*, applied in the shear test were 50 kPa, 100 kPa, 200 kPa, and 300 kPa. Thus, 28 sand samples needed to be made for each relative density. The main steps in the testing process were as follows:

(i) A preliminary verification as to whether the direct shear instrument and data acquisition system could operate normally was carried out. (ii) The circular container of the lower box of the shear box was removed, and the prepared concrete block was placed into the lower box of the shear box, as shown in Figure 4. (iii) The upper shear box was filled with sand in layers according to the specified relative density. (iv) Lastly, routine shear tests were conducted at a shear rate of 1.2 mm/min on the different interfaces.

## 3. Results and Discussion

### 3.1. Shear Stress–Displacement Relationship

**Figure 5:**
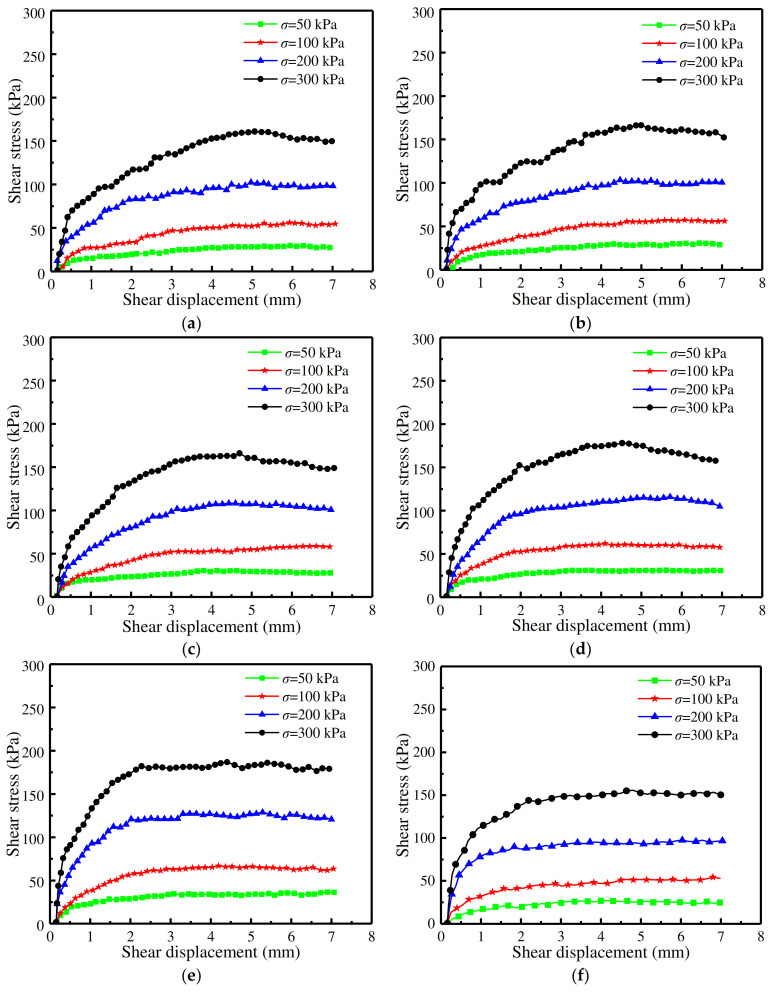
Shear stress–displacement curves with different normal stress values at a relative density of 30% with: (**a**) rectangular; (**b**) trapezoid; (**c**) semicircular; (**d**) triangular; (**e**) random; and (**f**) smooth roughness morphologies.

Figure 5 shows the shear stress–displacement curves with different interface morphologies at relative density of 30%, and the curve trend with a relative density of 50% was consistent with that of 30%. This indicates that the normal stress value significantly influenced the shear behavior of sand–rough concrete interfaces at the sand relative densities of 30% and 50%. When the normal stress value was 50 kPa, the curves exhibited virtually no elastic stage, instead directly entering the plastic deformation stage. When the normal stress value was 100 kPa or 200 kPa, the curves showed a slight strain hardening. Lastly, when the normal stress value was 300 kPa, the curve displayed a slight strain softening. However, when the relative density was 70%, the shear stress–displacement relationship revealed ideal elastoplasticity under the normal stress value of 300 kPa.

**Figure 6:**
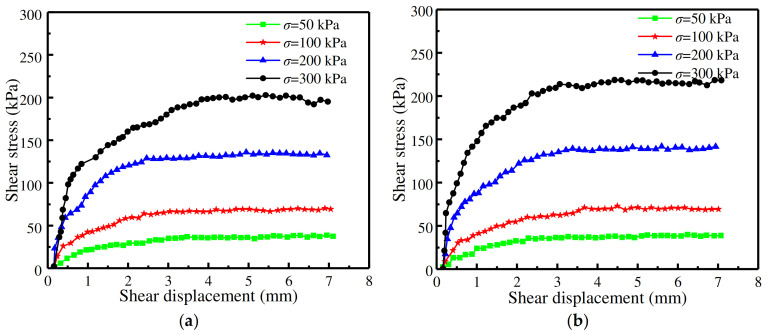
Shear stress–displacement curves with different normal stress values at a relative density of 50% and 70% with random roughness morphologies: (**a**) *D*r = 50%; (**b**) *D*r = 70%.

**Figure 7:**
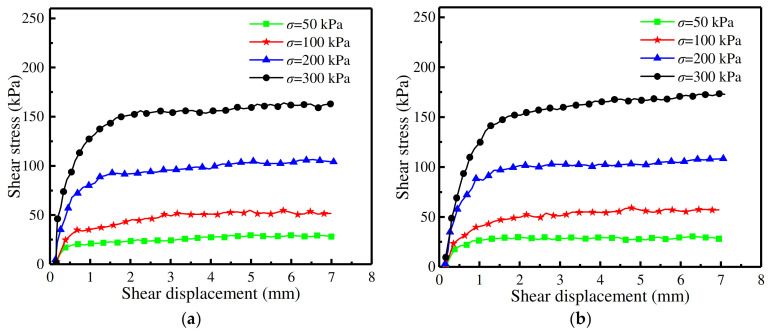
Shear stress-displacement curves with different normal stresses values at a relative density of 50% and 70% with smooth roughness morphologies: (**a**) *D*r = 50%; (**b**) *D*r = 70%.

In the cases of random rough concrete surfaces, the variation law of shear stress–displacement curves was found not to have been affected by the relative density and normal stress values, as evidenced by the virtual absence of any softening trend, as shown in Figure 5e and Figure 6. Moreover, as shown in Figure 5f and Figure 7, the shear stress–displacement curves of sand–smooth concrete interfaces with three relative density values displayed ideal elastoplasticity properties, which was in agreement with the results of Guo et al. (2020) [19] and Lu et al. (2013) [20].

### 3.2. Shear Strength

**Figure 8:**
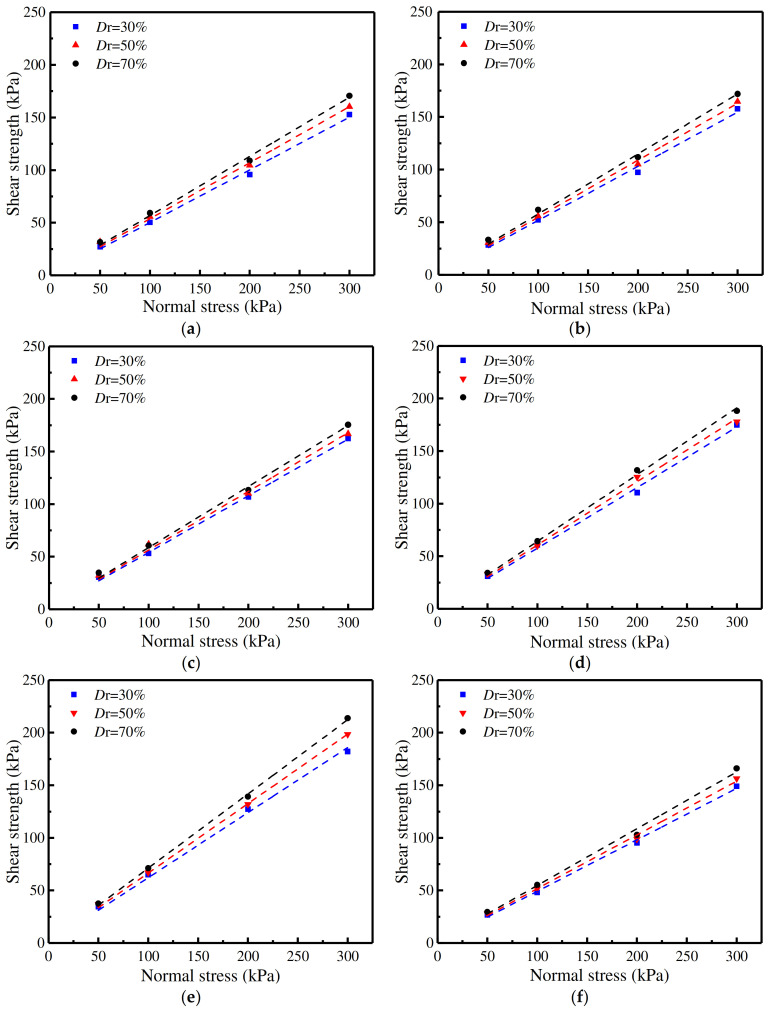
Shear strength curves with different relative densities with: (**a**) rectangular; (**b**) trapezoid; (**c**) semicircular; (**d**) triangular; (**e**) random; and (**f**) smooth roughness morphologies.

Figure 8 represents the shear strength curves of the concrete surfaces with the five rough morphology types and of the one smooth concrete surface. It upholds the consistent law that the shear strength of sand–concrete interfaces rises as the relative density increases. When the normal stress value was 50 kPa, the effect of the relative density on the shear strength of sand–concrete interfaces was not obvious; however, as the normal stress was augmented, the impact of the relative density was significant [21]. It can thus be seen from Figure 8 that the shear strength curves (the fitting curves) could be represented by straight lines, thereby establishing that the shear failure value of the sand–concrete interfaces conformed to the Mohr Coulomb strength criterion [22,23]. As it is generally well known that sand comprises very low cohesion values [17], the cohesion values of the five types of sand–concrete interfaces were all taken in such a manner as to fit with the cohesion value of the sand–smooth concrete interface. As the correlation coefficients of the fitting curves were above 0.996 (the closer the correlation coefficient is to 1, the better the fitting result is), the cohesion values were deemed reasonable. The shear strength indices and correlation coefficients with different concrete surface morphologies are shown in Table 4.

**Figure 9:**
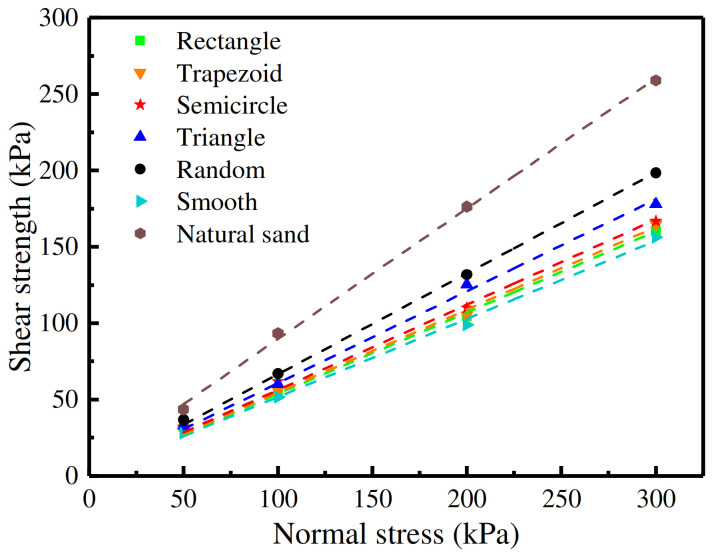
Shear strength curves with different interface morphologies at a relative density of 50%.

Figure 9 presents the shear strength curves of sand–concrete surfaces with different morphologies at a relative density of 50%. From Figure 9, the following descending order of shear strength can be observed: when the normal stress was constant, the shear strength of the sand was the greatest; in second place was the shear strength of the sand–random concrete interfaces, with the shear strength of the sand–smooth concrete interfaces being the lowest. The order of internal friction angles from the greatest to the smallest was: (i) natural sand, (ii) sand–random concrete interface, (iii) sand–triangular groove concrete interface, (iv) sand–semicircular groove concrete interface, (v) sand–trapezoid groove concrete interface, (vi) sand–rectangular groove concrete interface, and lastly (vii) sand–smooth concrete interface. It was, therefore, concluded that the shear strength of the triangular groove concrete interface most closely approximated the shear strength of the random concrete interface.

### 3.3. Shear Failure Mechanism

**Figure 10:**
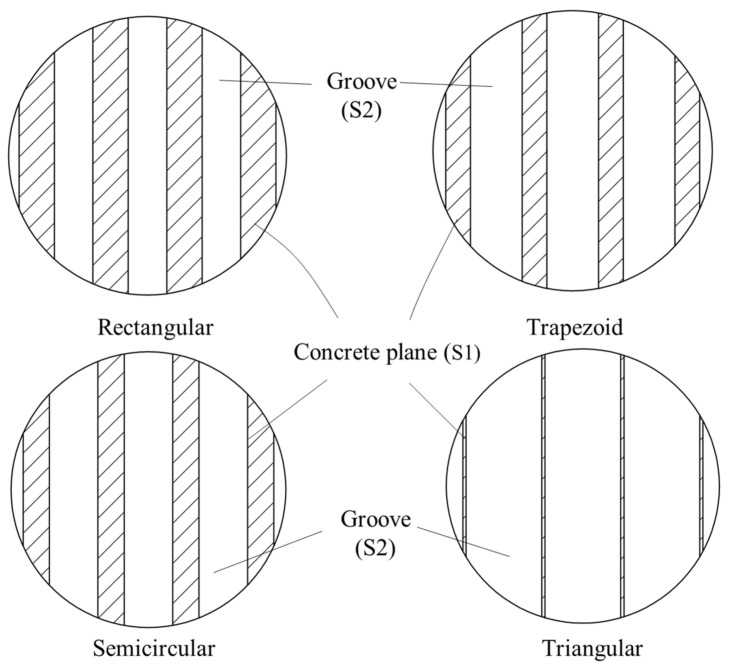
Contact surfaces between concrete plane and sand with different interface morphologies (the shaded parts are S1, which are the contact areas between concrete plane and sand; the areas of the sand sample are S2).

Figure 10 illustrates the contact surfaces between concrete plane and sand with different interface morphologies. As can be observed, the shaded portion is S1, which is the contact area between concrete plane and sand, while the area of the sand sample is S2. The area values of S1 and S2 and the ratio of S1 to S2 are listed in Table 5. The findings of the shear strength tests conducted were that the shear strength between the sand and the regular concrete surface in descending order was triangular, semicircular, trapezoid, and rectangular. From Figure 10 and Table 5, it can be seen that the shear strength of the sand–concrete interface was higher when the contact area between concrete plane and sand was smaller. The potential reasons for this result include the following: the direct shear tests were conducted along the fixed contact surface, which meant that the entire contact surface between sand and concrete could be divided into two sections, one being the contact area between sand and concrete plane, and the other, the sand filling the grooves of the concrete surface, which is equivalent to a contact between sand and sand. Therefore, it followed in the present authors’ view that the shear process could likewise be divided into two parts, namely (i) the shearing along the sand and concrete plane interfaces, and (ii) the equivalent of shearing in natural sand.

Previous studies had shown that while the shear strength of interfaces rose with the increase in interface roughness, this augmentation remained less than the internal shear strength of sand [24,25], while the shear strength of the sand–smooth concrete interface proved to be the smallest [26]. Accordingly, when the contact area between sand and concrete plane was smaller, a greater shear area occurred in the natural sand, resulting in a higher shear strength. In the case of the random concrete surface, the contact area between sand and concrete plane was very limited—sand particles were mainly embedded in random grooves of the concrete. The shear displacement was found to have caused the sand particles either to roll or “bite” along the concrete groove or to shear in the natural sand. Therefore, the shear strength of the random concrete surface was greater than that of the regular concrete surfaces.

## 4. Conclusions

The shear mechanical properties of the soil–structure contact surface are a keen research topic in geotechnical engineering, and the previous studies have mainly focused on the effect of concrete surface roughness on interface shear mechanical properties by constructing concrete blocks with regular rough surfaces. However, the morphology of the contact surface between the cast-in-place concrete structure and the surrounding soil in engineering is random, and the morphology of regular rough surfaces constructed in different studies also varies. Therefore, it was necessary to study the potential impact of concrete surfaces with various roughness morphologies on interface mechanical properties. In this study, based on the roughness value of a random concrete surface, four regular types of concrete specimen contact surfaces with the same roughness but different groove morphologies were produced, as well as a smooth concrete surface sample. A series of direct shear tests were performed. The relationship between shear stress and shear displacement during the shear process was analyzed. The effects of the interface morphology on the shear strength of the sand–concrete interfaces were investigated, and the main conclusions were as follows: (1) The rough morphology of concrete surface had little effect on the variations in the shear stress–displacement curves of the sand–concrete interfaces. (2) The shear strength of the sand–concrete interfaces varied with the differences in concrete surface morphology, and the shear strength of the concrete surface with random rough morphology was proved to be the greatest. (3) Among the concrete surfaces with regular rough morphologies, the shear strength of the triangular groove concrete surface was the highest, which was closest to the shear strength of the random rough concrete surface, while the shear strength of the rectangular groove concrete surface proved to be the lowest. (4) The shear strength of sand–concrete interfaces with the same roughness value was affected by the size of the contact area between the concrete plane and sand. The greater the contact area between the concrete plane and the sand, the lower the shear strength of the sand–concrete interface was found to be.

There are currently numerous evaluation methods of roughness, among which the sand-cone evaluation method was selected for this study. The average depth of the random concrete surface was the same as that of the four regular concrete surfaces (rectangular, trapezoid, semicircular, and triangular). The results in the present research showed that different contact surface types with the same roughness value yielded different shear strength values. It was thus inferred that solely evaluating concrete surface roughness on the basis of its average depth may not reflect all of the properties of the concrete surface in question. Accordingly, the present authors concluded that in the future, rough concrete surfaces should be constructed on the basis of other roughness evaluation methods to further investigate the properties of the sand–concrete interfaces. In addition, further research is also needed on roughness evaluation methods, so that the research results obtained can be better applied to engineering practice.

## Figures and Tables

**Table 1:** Basic physical properties of the natural river sand used in the tests.

Property	Value
Specific gravity (*G*_s_)	2.65
*D*_10_ (mm)	0.30
*D*_30_ (mm)	0.61
*D*_60_ (mm)	1.39
Uniformity coefficient (*C*_u_)	4.63
Coefficient of curvature (*C*_c_)	0.89
Maximum void ratio *e*_max_	1.04
Minimum void ratio *e*_min_	0.57

**Table 2:** Dimensional parameters of different concrete surface morphologies.

Groove Type	*L* (mm)	*H* (mm)	*l*_1_ (mm)	*l*_2_ (mm)	*l*_3_ (mm)	*h*_1_ (mm)
Rectangular	96	20	8.57	7.86	10.86	5.46
Trapezoid	96	20	11.43	5.48	8.47	5.46
Semicircular	96	20	10.92	5.9	8.9	5.46
Triangular	96	20	17.14	0.72	3.71	5.46

**Table 3:** Group comparison table for shear test.

Specimen	S_1	S_2	S_3	S_4	S_5	S_6	S_7
Test object	Natural sand	Sand–concrete (smooth surface)	Sand–concrete (random surface)	Sand–concrete (Triangular groove surface)	Sand–concrete (Rectangular groove surface)	Sand–concrete (Trapezoid groove surface)	Sand–concrete (Semicircular groove surface)
Normal stress (kPa)	50, 100, 200, 300						

**Table 4:** Shear strength indices with different concrete surface morphologies.

*D*r (%)	Interface Morphology	Shear Strength Formula	*c* (kPa)	*φ* (°)	R
30	Smooth	*τ*_f_ = 0.1522 + 0.4896*σ*	0.1522	26.09	0.996
	Rectangular	*τ*_f_ = 0.1522 + 0.5001*σ*	0.1522	26.57	0.998
	Trapezoid	*τ*_f_ = 0.1522 + 0.5147*σ*	0.1522	27.23	0.998
	Semicircular	*τ*_f_ = 0.1522 + 0.5388*σ*	0.1522	28.32	0.999
	Triangular	*τ*_f_ = 0.1522 + 0.5757*σ*	0.1522	29.93	0.998
	Random	*τ*_f_ = 0.1522 + 0.6190*σ*	0.1522	31.76	0.998
	Natural sand	*τ*_f_ = 4.4300 + 0.8188*σ*	4.4300	39.31	0.993
50	Smooth	*τ*_f_ = 0.5724 + 0.5111*σ*	0.5724	27.07	0.996
	Rectangular	*τ*_f_ = 0.5724 + 0.5320*σ*	0.5724	28.01	0.998
	Trapezoid	*τ*_f_ = 0.5724 + 0.5414*σ*	0.5724	28.43	0.999
	Semicircular	*τ*_f_ = 0.5724 + 0.5576*σ*	0.5724	29.14	0.998
	Triangular	*τ*f = 0.5724 + 0.6017*σ*	0.5724	31.04	0.999
	Random	*τ*_f_ = 0.5724 + 0.6599*σ*	0.5724	33.42	0.999
	Natural sand	*τ*_f_ = 4.3263 + 0.8535*σ*	4.3263	40.48	0.998
70	Smooth	*τ*_f_ = 0.6425 + 0.5400*σ*	0.6425	28.37	0.993
	Rectangular	*τ*_f_ = 0.6425 + 0.5619*σ*	0.6425	29.33	0.999
	Trapezoid	*τ*_f_ = 0.6425 + 0.5709*σ*	0.6425	29.72	0.998
	Semicircular	*τ*_f_ = 0.6425 + 0.5803*σ*	0.6425	30.13	0.998
	Triangular	*τ*_f_ = 0.6425 + 0.6352*σ*	0.6425	32.42	0.999
	Random	*τ*_f_ = 0.6425 + 0.7055*σ*	0.6425	35.20	0.999
	Natural sand	*τ*_f_ = 4.7900 + 0.9084*σ*	4.7900	42.25	0.995

**Table 5:** Contact area values between concrete surface and sand.

Groove Type	S1/mm^2^	S2/mm^2^	S1/S2
Rectangular	1473.00	3000	49.11%
Trapezoid	1019.89	3000	34.00%
Semicircular	1000.20	3000	33.34%
Triangular	129.49	3000	4.32%

## Data Availability

All data supporting the results of this study are included within the article.

## References

[1] Hu L.M., Pu J.L. (2004). Testing and Modeling of Soil-Structure Interface. J. Geotech. Geoenvironmental Eng..

[2] Isaev O.N., Sharafutdinov R.F. (2020). Soil Shear Strength at the Structure Interface. Soil Mech. Found. Eng..

[3] Beren M., Çobanoglu I., Çelik S., Ündül Ö. (2020). Shear Rate Effect on Strength Characteristics of Sandy Soils. Soil Mech. Found. Eng..

[4] Uesugi M., Kishida H. (1986). Influential Factors of Friction Between Steel and Dry Sands. Soils Found..

[5] Zhang M., Sang S., Wang Y., Bai X. (2020). Factors Influencing the Mechanical Characteristics of a Pile–Soil Interface in Clay Soil. Front. Earth Sci..

[6] Martinez A., Palumbo S., Todd B.D. (2019). Bioinspiration for Anisotropic Load Transfer at Soil–Structure Interfaces. J. Geotech. Geoenvironmental Eng..

[7] Wang X., Wang X.Z., Zhu C.Q., Meng Q.S. (2019). Large-scale Direct Shear Tests of Interfaces Between Different Soils and Concrete Considering Roughness Effect. Adv. Eng. Sci..

[8] Li M.Y., Li Y.H., Islam M.R. (2021). Effects of water content and interface roughness on the shear strength of silt–cement mortar interface. Soils Found..

[9] Vafaei N., Fakharian K., Sadrekarimi A. (2021). Sand-sand and sand-steel interface grain-scale behavior under shearing. Transp. Geotech..

[10] Li Y.H., Lv M.F., Guo Y.C., Huang M.S. (2021). Effects of the soil water content and relative roughness on the shear strength of silt and steel plate interface—ScienceDirect. Measurement.

[11] Koval G., Chevoir F., Roux J.-N., Sulem J., Corfdir A. (2011). Interface roughness effect on slow cyclic annular shear of granular materials. Granul. Matter.

[12] Maghsoodi S., Cuisinier O., Masrouri F. (2020). Thermal effects on mechanical behaviour of soil–structure interface. Can. Geotech. J..

[13] Haeri H., Sarfarazi V., Zhu Z., Marji M.F., Masoumi A. (2019). Investigation of shear behavior of soil-concrete interface. Smart Struct. Syst..

[14] Wang Y.B., Zhao C., Wu Y. (2020). Study on the Effects of Grouting and Roughness on the Shear Behavior of Cohesive Soil-Concrete Interfaces. Materials.

[15] Martinez A., Frost J.D. (2017). The influence of surface roughness form on the strength of sand–structure interfaces. Géotechnique Lett..

[16] Wang X., Cheng H., Yan P., Zhang J., Ding Y. (2020). The influence of roughness on cyclic and post-cyclic shear behavior of red clay-concrete interface subjected to up to 1000 cycles. Constr. Build. Mater..

[17] Li H., Fu S., Zhu D., Li G., Shen S. (2023). Experimental study on the effects of triangular groove inclination angles on the mechanical behavior of sand–concrete interfaces. J. Mater. Res. Technol..

[18] (2019). Standard for Geotechnical Testing Method. Ministry of Water Resources of the People’s Republic of China.

[19] Guo J., Wang X., Lei S., Wang R., Kou H., Wei D. (2020). Effects of Groove Feature on Shear Behavior of Steel-Sand Interface. Adv. Civ. Eng..

[20] Lu Y., Zhou G.Q., Xia C.H., Wang P.S. (2013). Effect of shape scale on characteristics of coarse grained soil-structural interface under medium and high pressures. Rock Soil Mech..

[21] Janipour A.K., Mousivand M., Bayat M. (2022). Study of interface shear strength between sand and concrete. Arab. J. Geosci..

[22] Zhao C.F., Gong H., Zhao C. (2012). Elastoplastic analysis of interface between clay and concrete considering effect of normal stress history. Chin. J. Rock Mech. Eng..

[23] Chen J.H., Zhang J.S., Li J. (2016). Experimental research on mechanical characteristics of cohesive soil-structure interface by considering its roughness. J. Cent. South Univ. (Sci. Technol.).

[24] Su L.J., Zhou W.H., Chen W.B., Jie X. (2018). Effects of relative roughness and mean particle size on the shear strength of sand-steel interface. Measurement.

[25] Wang N., Zhang J., Wang Y., Zhang H., Ma Y., Zhao L., Guo Q. (2020). Experimental Study on Mechanical Properties of Grout–Soil Interface in Anchor System of Rammed Earthen Sites. Int. J. Géoméch..

[26] Chen X., Zhang J., Xiao Y., Li J. (2015). Effect of roughness on shear behavior of red clay—concrete interface in large-scale direct shear tests. Can. Geotech. J..

